# Long-term outcome of ovarian function after drug-free *in vitro* activation (IVA) in primary ovarian insufficiency patient

**DOI:** 10.5935/1518-0557.20200093

**Published:** 2021

**Authors:** Janisse Ferreri, Marta Méndez, José Maria Calafell, Francesc Fábregues

**Affiliations:** 1 Institut Clinic of Gynecology, Obstetrics and Gynecology. Hospital Clinic of Barcelona, Barcelona, Spain

**Keywords:** Akt stimulation, Hippo signaling, ovarian failure, pregnancy, surgical in vitro activation

## Abstract

Drug-Free IVA has been recently introduced as a therapeutic option for patients with Primary Ovarian Insufficiency (POI). Despite the existing limited results, it can be considered as a promising option for these patients to achieve their own offspring. Here we report the case of a 35-year-old woman diagnosed with POI at 30 years of age. Drug-Free IVA was performed at age 33 and pregnancy was achieved by IVF 10 months after grafting. Unfortunately, she had a preterm delivery with neonatal death due to prematurity complications. After delivery, she recovered spontaneous ovarian function and one mature oocyte was retrieved 20 months after Drug-Free IVA. Following IVF, one embryo was transferred, and she is currently 33 weeks pregnant, suggesting that Drug-free IVA could lead to long-term ovarian function.

## INTRODUCTION

Primary ovarian insufficiency (POI) affects 1% of women, and it is characterized by high circulating FSH levels, together with amenorrhea before 40 years of age (Nelson, 2009). These patients are infertile due to a lack of follicle growth and ovulation. Although menstrual cycles cease in these patients, some of them still contain residual small ovarian follicles, which do not produce enough circulating estrogens and progesterone to modulate uterine functions. Nowadays, the only chance for bearing a baby is through egg donation (De Vos *et al*., 2010).

A method for activating dormant follicles in the mammalian ovary was proposed and named *ovarian in vitro activation* (IVA). Competent, fertilizable oocytes and healthy babies were obtained from residual follicles with the IVA approach (Hsueh *et al*., 2015; Suzuki *et al*., 2015). Recently, it has been proposed that secondary follicle growth could be restored using IVA simplified by carrying out ovarian tissue fragmentation alone, without the need to tissue culture, in a single surgical act known as Drug-Free IVA (Ferreri et al, 2020; Fàbregues et al., 2018; Kawamura et al., 2020). According to this concept, with this technique modification, ovarian follicular activation should be recovered a few months after surgery. Although, its long-term efficacy still needed to be further assessed.

Here we present the case of a patient with POI in whom ovarian activation was maintained 20 months after performing a Drug-free IVA.

### Case description

Our patient is a 35-year-old woman with POI diagnosed 5 years before. She fulfilled all three diagnosis criteria from the ESHRE POI guideline: less than 40 years of age, 3-4 months of amenorrhea (she already experienced 3 years of continuous amenorrhea) and had > 25 IU/l on two occasions > 4 weeks apart (initial FSH levels of 78.3 and 57.2 UI/L). After an extensive clinical and analytical assessment, all known etiologies of POI were ruled out (chromosomal and genetic defects; autoimmune ovarian damage; environmental causes; infectious causes or iatrogenic causes) and she was diagnosed with idiopathic POI (European Society for Human Reproduction and Embryology (ESHRE) Guideline Group on POI, 2016).

She was recruited to our Drug-Free IVA research after meeting the inclusion criteria (which also includes AMH <0.1 ng/mL and zero Antral Follicular Count, AFC = 0). After providing written informed consent approved by The Ethics Research Committee of the Hospital Clinic of Barcelona, she was successfully included in our study.

At the Drug-Free IVA surgery preparation, she was 33 years old, normal BMI (23.6 Kg/m^2^) and had an unremarkable clinical history. Noteworthy, she had her menarche at 13 years of age, and as early as two years later, she started having irregular cycles, and became continuously amenorrhoeic in her late 30 years of age.

Following our study protocol one month before surgery, pretreatment with oral estrogen/progesterone (Progyluton; Bayer, Spain) was performed in an attempt to suppress serum gonadotropin levels, and it was stopped the day before surgery.

**Figure 1:**
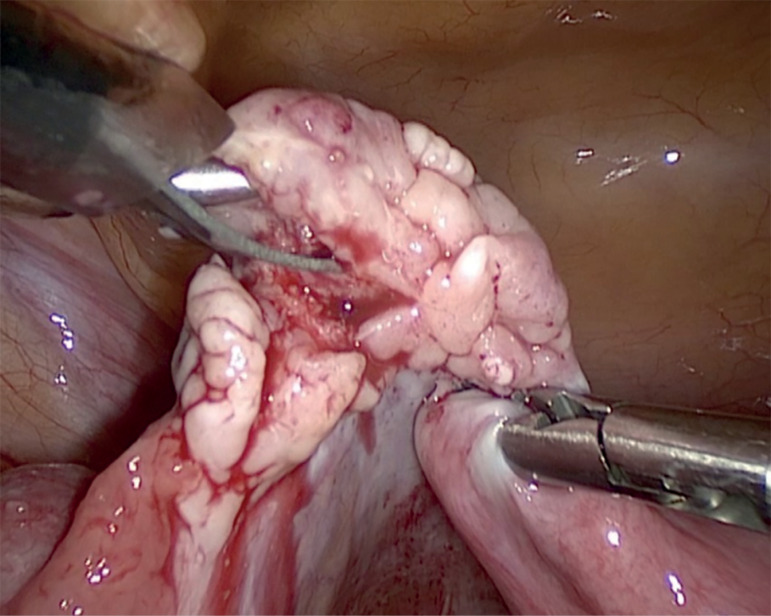
Laparoscopic extraction of ovarian cortex

**Figure 2:**
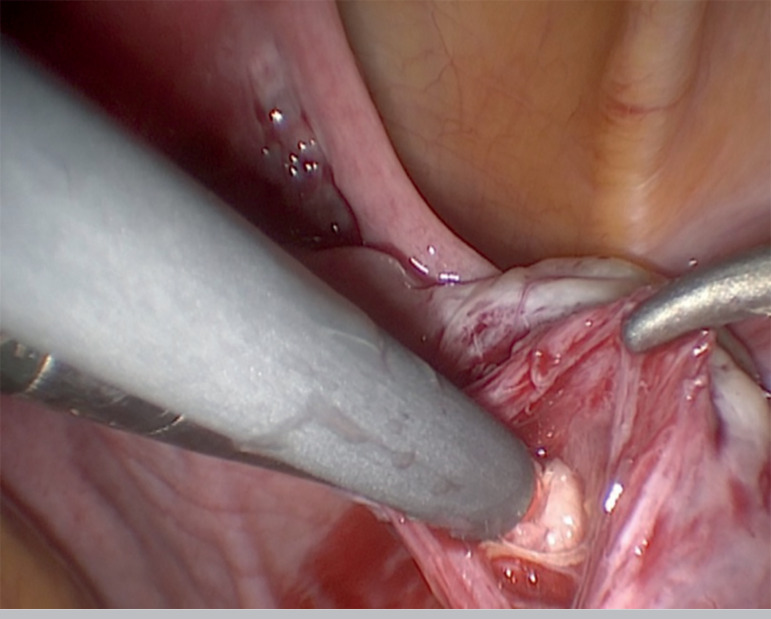
Placement of ovarian fragments in the peritoneal pocket near the contralateral ovary

**Figure 3:**
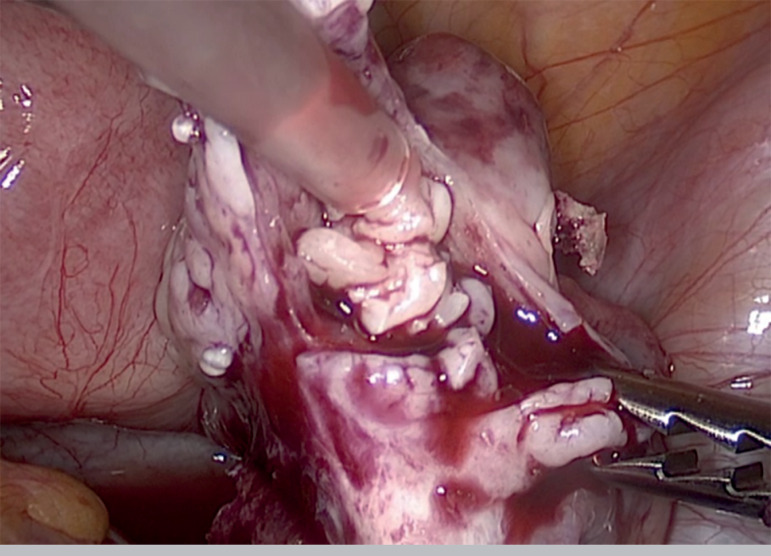
Placement of ovarian fragments in the contralateral ovary

Minimally invasive laparoscopic approach was carried out. The surgical procedure included laparoscopic removal of the right ovarian cortex, fragmentation of tissue and autografting, all in a single surgical act (Drug-Free IVA) (Fàbregues *et al.*, 2018; Ferreri *et al*., 2020). Briefly, as previously published, ovarian tissue was removed, using scissors to minimize trauma to the sensible ovarian cortex, and meticulous bipolar coagulation in the remaining medulla. Two-thirds of the ovarian cortex were released, than the medulla was extracted by dissection with small scissors to obtain thin layers of ovarian cortex fragments. Auto-transplantation and ovarian tissue graft were performed in the contralateral ovary and in the peritoneal pocket near the ovary, depositing 10-12 fragments of ovarian tissue in each surgical site. We used N hexyl-2 cyanoacrylate as a fixation surgical treatment in the transplantation site during ovarian tissue transplantation, in order to avoid sutures (Fig. 1,2,3). The patient was discharged the same day of the surgery with no complications. One of the ovarian fragments was used for histological analysis, to determine the presence of residual follicles: no residual follicle were found.

Hormone stimulation was started 4 days after surgery in two batches of 21 days of daily purified urinary HMG (300 IU) (Meriofert; Angelini, Spain) together with GnRH agonist (triptorelin, 0.1 mg/d) (Decapetyl; Ipsen Pharma, Spain) separated by 2 months of daily oral estrogen/progesterone (Progyluton; Bayer, Spain). We abandoned the treatment because there was no response during the first two stimulated cycles.

As provided in the study protocol, monthly clinical follow-up was established, and in the tenth month after Drug-Free IVA, the patient started regular spontaneous menstrual cycles. We performed serial pelvic sonograms and estradiol analyses to follow up on her follicular wave. When the follicle achieved 18 mm, recombinant hCG (Ovitrelle 250, Merck, Spain) was administered, and thirty-six hours later oocyte retrieval was performed. One mature oocyte was retrieved, fertilized through ICSI and day 3 single embryo was transferred. She had a singleton pregnancy.

Her pregnancy underwent normally with a correct first trimester screening for chromosomal abnormalities. Unfortunately, cervical insufficiency was diagnosed in the twenty-third week of pregnancy and despite emergency cervical cerclage; she had preterm delivery with neonatal death due to prematurity complications. Six weeks after delivery she recovered spontaneous ovarian function again. After 2 months, 20 months from Drug-Free IVA, the patient fully recovered and we restarted monthly follow-up, as described before, pursuing follicular wave.

When her follicle achieved triggering criterion, recombinant hCG (Ovitrelle 250, Merck, Spain) was administered, and thirty-six hours later oocyte retrieval was performed. This time, one mature oocyte was retrieved, also fertilized through ICSI and a day 3 single embryo was transferred. A second singleton pregnancy was established. Currently, specialized obstetrician control is ongoing in our prematurity unit and now, she is 33 weeks pregnant.

## DISCUSSION

The pool of primordial follicles in the ovary decreases dramatically with aging. In the case of POI, ovarian function ceases before the age of 40. However, it has been known for a long time that there are still residual (dormant) follicles in these ovaries. Different approaches to regenerate, rejuvenate or reactivate germ cells in the human ovary have been unsuccessful reported in the past.

Recently, physiological aspects that have provided data on the gonadotropin-independent phases of folliculogenesis have been described. Concretely, it has been showed that the balance between activating (Akt stimulatory) and inhibitory pathways (Hippo signaling), are crucial, adequate and proportional to follicular activation (Hsueh *et al*., 2015). In this same line, it has been demonstrated that the manipulation of these indicated pathways, can have clinical application: (i) the disruption of the Hipposignaling pathway by fragmenting ovarian tissue and (ii) activating Akt pathway by incubation of ovarian tissue with stimulant substance in primary ovarian insufficiency patients (POI) (Kawamura *et al*., 2016; Suzuki *et al*., 2015). This procedure was named ovarian in vitro activation (IVA).

Some aspects have hampered the wide use of this technique. On the one hand, the need to perform two laparoscopies and, on the other, the possible harmful effects of stimulant substances (Griesinger & Fauser , 2020). In this way, very recently a modification of the technique has been reported, aiming at focusing only on the disruption of the hippo signaling pathway, performing the removal of the ovarian cortex, and auto transplantation by laparoscopy in the same surgical act, renouncing the chemical activation of the ovarian tissue (Drug-Free IVA) (Fàbregues *et al*., 2018, Kawamura *et al*., 2020; Ferreri *et al*., 2020).

Considering the established concepts on the effect of the Hippo signaling pathway disruption in the early stages of folliculogenesis, the Drug-Free IVA technique would only be able to achieve the activation of secondary follicles, and therefore could not activate primordial follicles, showing a possible limitation of its activation capacity (Hsueh *et al*., 2015).

Earlier studies have demonstrated that FSH receptors are expressed in follicles from primary to later stages and, together with the FSH and LH treatments; it promotes preantral follicle growth (Hsueh *et al*., 2015). In this line, we used higher HMG doses but, there could be spontaneous follicular waves. In fact, spontaneous follicular waves have already been objectified (Ferreri *et al*.,2020). The case we present here shows for the first time that the Drug-Free IVA technique has been able to maintain ovarian follicular activation for a long time, since, as observed in our patient, follicular waves were observed up to 20 months after surgery.

Obviously, more studies are needed to understand aspects associated with the true efficacy of ovarian activation in POI patients. However, the recently reported results, and specifically the clinical case reported here, represent a promising advance in the reproductive options of patients with POI, based on what is possible to activate the remaining “ovarian follicular gold reserve”.
